# Effect of heating insufflation tube of AirSeal system on laparoscopic surgery

**DOI:** 10.1038/s41598-023-50321-y

**Published:** 2024-01-05

**Authors:** Gumpei Yoshimatsu, Kurumi Sahara, Ryo Ohno, Ryuji Kajitani, Taro Munechika, Yoshiko Matsumoto, Hideki Nagano, Toshifumi Watanabe, Naoya Aisu, Yoichiro Yoshida, Suguru Hasegawa

**Affiliations:** 1 Department of Gastroenterological Surgery, Fukuoka University Hospital, 7-45-1 Nanakuma Jonan-Ku, Fukuoka, 814-0180 Japan; 2 Department of Regenerative Medicine and Transplantation, Fukuoka University, 7-45-1 Nanakuma Jonan-Ku, Fukuoka, Japan

**Keywords:** Gastroenterology, Medical research, Oncology

## Abstract

The AirSeal system (CONMED, NY, USA) can outstandingly keep pneumoperitoneum stable. However, water droplets form on the access port, impairing the performance of comfortable surgical procedures because of the resultant wet surgical field. This study was performed to clarify the mechanism of water droplet formation and to prevent it. Condensation was observed on the AirSeal system. A heater was wrapped around the tri-lumen tube, and the heating effect was assessed. The simulator experiments revealed that condensation formed in the tri-lumen tube and on the wall of the access port. The accumulated weight of the condensation on the wall of the access port was 41.6 g in the Heated group, 138.2 g in the Control group, and 479.4 g in the Cooled group. In the clinical assessment, the accumulated volume of the condensation attached to the inside wall was significantly smaller in the Heated group than in the Unheated group (111.7 g vs. 332.9 g, respectively). We clarified that the volume of condensation attached to the wall of the access port depended on the temperature of the tri-lumen tube. The clinical study revealed that the condensation on the access port was reduced by heating the tri-lumen tube. The development of a novel heating device for the insufflation tube would be effective and useful.

## Introduction

During the past two decades, the number of laparoscopic surgeries has markedly increased with the development of medical devices and minimally invasive surgery. In particular, the carbon dioxide insufflator is the most essential device for creating a large working space and clear view to conduct precise and sophisticated laparoscopic procedures.

The AirSeal system (CONMED, Utica, NY, USA) is a unique insufflation system developed as a valveless trocar device. The access port of the AirSeal system has no physical valve structure to maintain the intra-abdominal pressure and intraperitoneal surgical space. Instead, recirculated gas through the tri-lumen tube creates a cap on the top of the access port, termed the “air seal barrier,” and prevents intraperitoneal gas from leaking out during insufflation. When the intra-abdominal pressure exceeds the appropriate intraperitoneal pressure, the intraperitoneal gas leaks out through the air seal barrier to keep the intraperitoneal pressure within the appropriate range^1^. The AirSeal system reportedly helps to reduce shoulder pain due to excessive intraperitoneal pressure during laparoscopic surgery compared with the conventional insufflator, which has a mechanical valve on the access port^2–4^. Moreover, this system shortens the operative time because it constantly maintains a large intra-abdominal space for surgical procedures^5^.

However, the AirSeal system also has the disadvantage of causing a moist surgical field. This can be problematic during surgery, especially when lymph nodes are dissected around major blood vessels and tissue is mobilized. Therefore, it is important to elucidate the mechanism of this condensation formation in the AirSeal system to effectively manage the condensation and allow the performance of sophisticated surgery. In this study, we explored the mechanisms underlying the formation of condensation on the access port and aimed to reduce this condensation to facilitate the comfortable performance of surgical procedures.

## Results

### Increased condensation in tri-lumen tube and access port during AirSeal insufflation in simulator model

We observed the tri-lumen tube and access port to identify the mechanism of condensation formation during AirSeal insufflation. First, we determined that the condensation arose in one of the lumens of the tri-lumen tube, especially within the tube creating the air seal barrier through the recirculated gas from the abdomen. Second, we found that the condensation was attached to the inside and outside walls of the access port. The tri-lumen tube consists of three tubes. One tube provides insufflation from a tank to the abdomen through the body of the AirSeal. This insufflated gas is dry but cooler than body temperature, and it cools the access port and neighboring tubes. The second tube withdraws the excess gas from the abdomen to the body of the AirSeal. This collected gas includes moisture from the abdominal cavity. The third tube creates an air seal barrier in the access port using the collected and recirculated gas from the abdominal cavity. The recirculated gas is moist because it is carried over from the abdominal cavity through the second tube. When the moist gas is cooled by the surrounding atmosphere or the neighboring tube due to the gas from the tank, condensation develops within the tube (Supplemental Video 1a). The condensation accumulates and eventually changes to water droplets, which flow to the inside of the access port (Supplemental Video 1b). Condensation simultaneously forms on the outside of the access port because of the decreasing temperature of the moist intra-abdominal gas (Supplemental Video 1c).

### Dependency of condensation volume on temperature of insufflation tube

**Figure 1:**
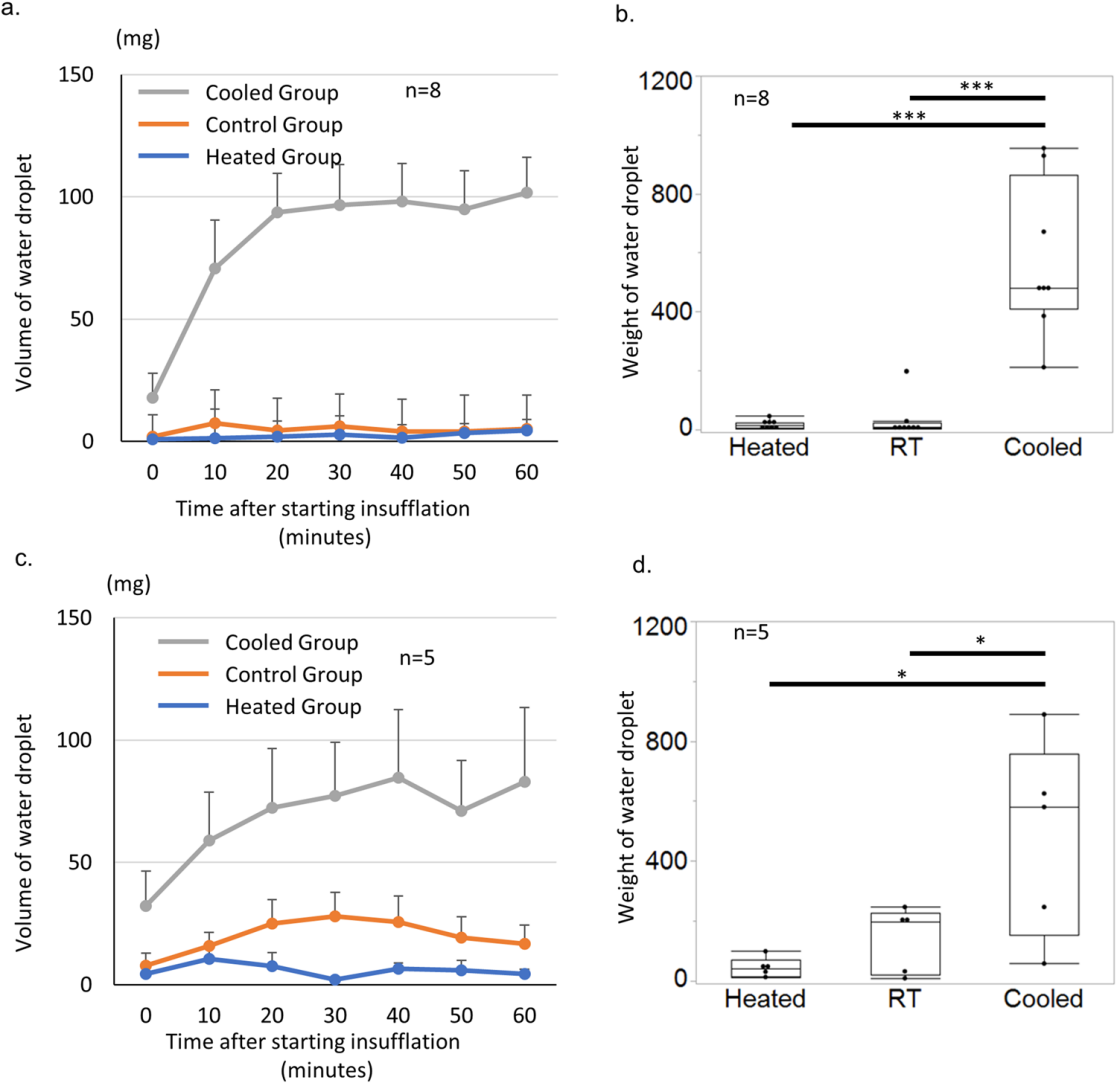
Measurement of condensation volume on access port in simulator model. The experiment was performed using the abdominal simulator and different temperatures of the tri-lumen tube (4 °C, room temperature, and 37 °C). The volume of the condensation on the inside and outside of the wall of the access port was measured every 30 min for 2 h in the each of the three groups and compared among the groups (i.e., Control, Cooled, and Heated groups). (**a**) The condensation volume on the inside wall of the access port was lower in the Heated and Control groups than in the Cooled group. (**b**) The accumulated weight of the condensation on the inside wall of the access port was significantly lower in the Heated and Control groups than in the Cooled group. (**c**) The condensation volume attached to the inside wall of the access port was lower in the Heated group than in the Cooled and Control groups. (**d**) The accumulated weight of the condensation on the inside wall of the access port was lower in the Heated group than in the Cooled and Control groups.

To clarify the influence of the temperature of the tri-lumen tube on the condensation attached to the inside and outside walls of the access port, an experiment was performed to assess the influence of the temperature in the tri-lumen tube using an abdominal simulator. The volume of condensation attached to the inside and outside of the wall of the access port was measured every 30 min for 2 h in the three aforementioned groups (Heated, Cooled, and Control groups) and then compared among the groups. Inside the access port, the weight trend of the condensation was significantly lower in the Heated and Control groups than in the Cooled group (p < 0.0001 for each. Figure 1a). The condensation gradually increased in the Cooled group for the first 30 min; after the 30-min time point, 100 mg of condensation developed on the inside wall of the port every 10 min. However, condensation did not form on the inside wall of the port in the Control and Heated groups. The accumulated weight of the condensation on the inside wall of the access port was significantly lower in the Heated and Control groups than in the Cooled group (Heated group vs. Control group vs. Cooled group: 16.5 ± 4.9, 33.0 ± 23.6, and 573.9 ± 92.1 g, respectively; Heated group vs. Cooled group: p < 0.0001 and Control group vs. Cooled group: p < 0.0001. Figure 1b).

In the Cooled group, condensation increasingly formed on the outside of the access port for the first 40 min; after this time point, 80 mg of condensation developed every 10 min. In the Control group, 20 mg of condensation formed on the outside of the access port every 10 min. However, only 10 mg of condensation formed every 10 min in the Heated group (Heated group vs. Control group: p = 0.0981 and Heated group vs. Cooled group: p = 0.0181. Figure 1c). The accumulated weight of condensation was significantly lower in the Heated than Cooled group and significantly lower in the Control than Cooled group (Heated group vs. Control group vs. Cooled group: 41.6 ± 15.4, 138.2 ± 49.3, and 479.4 ± 147.0 g, respectively; Heated group vs. Cooled group: p = 0.0125 and Control group vs. Cooled group: p = 0.0487. Figure 1d). These results suggest that the volume of the condensation attached to the access port depends on the temperature of the insufflation tube. In other words, condensation would be prevented by heating the insufflation tube.

### Reduction of intraoperative condensation volume on access port by heating of insufflation tube

**Figure 2:**
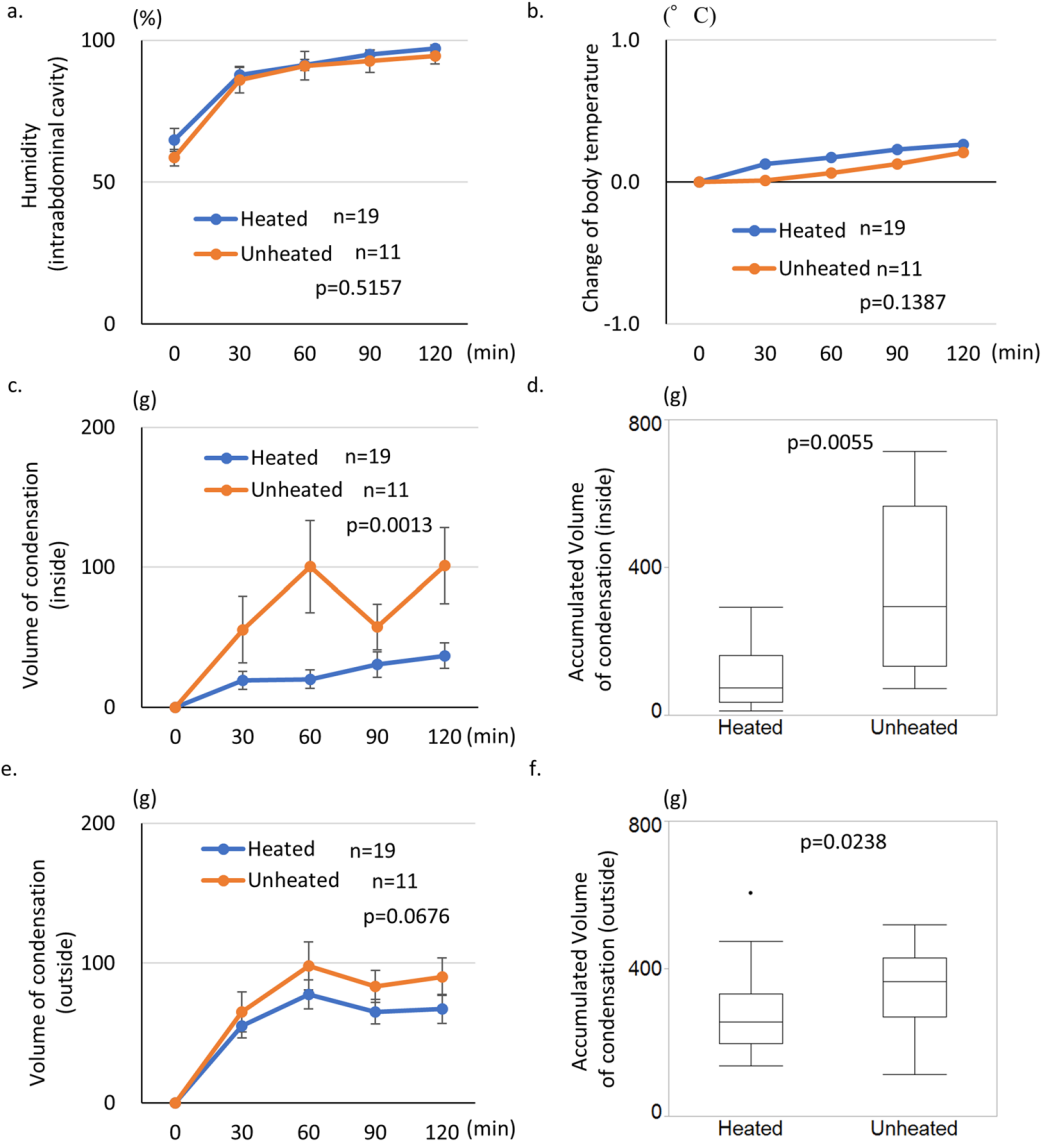
Measurement of condensation volume on access port during laparoscopic surgery. The condensation was wiped by gauze, and the condensation weight on the access port was measured using a scale to confirm the heating effect. (**a**) The humidity of the intra-abdominal cavity was measured during surgery, and both groups showed a similar trend. (**b**) The change in the patient’s body temperature was not significantly different between the two groups. (**c**) The volume of condensation attached to the inside wall of the access port demonstrated a significantly lower trend in the Heated group during surgery than in the Control group. (**d**) The accumulated condensation weight on the inside wall of the access port was significantly lower in the Heated group than in the Control group. (**e**) The volume of condensation attached to the outside wall demonstrated a slightly lower trend in the Heated group during surgery than in the Control group. (**f**) The accumulated condensation weight on the outside wall was significantly lower in the Heated group than in the Control group.

Thirty patients with right-sided colon cancer were enrolled in the study to clarify the effect of heating the tri-lumen tube on reducing the condensation attached to the access port (Table 1). The 30 patients were classified into the Unheated group (n = 11) and the Heated group (n = 19). The patients comprised more men than women, but the tumor location and TNM classification were not significantly different between the two groups. All surgeries were performed laparoscopically (laparoscopic ileocecal resection, right hemicolectomy). The intra-abdominal humidity was measured for the first 2 h after starting insufflation from the AirSeal system in the AirSeal mode. The humidity increased for 30 min after starting the AirSeal system, after which almost complete saturation was attained in both groups (p = 0.5157. Figure 2a). The body temperature did not substantially change during surgery (p = 0.1387. Figure 2b). The condensation volume was also measured during surgery, and the intra-abdominal humidity and body temperature were measured until 2 h after starting insufflation. Condensation appeared on both the inside and outside wall of the access port in the first 30 min (Fig. 2c). The weight of condensation on the inside wall gradually increased in the Unheated group as the surgery progressed, reaching 100.4 ± 32.9 g. In the Heated group, however, the weight of condensation attached to the inside wall gradually increased until 120 min after starting the AirSeal system, reaching a maximum of only 36.8 ± 9.1 g (Heated group vs. Unheated group: p = 0.0013). The accumulated volume of condensation attached to the inside wall was significantly smaller in the Heated group than in the Unheated group (111.7 ± 20.2 vs. 332.9 ± 74.1 g, respectively; p = 0.0055. Figure 2d). Regarding the condensation attached to the outside wall, both groups showed a similar trend during the first 2 h after starting the AirSeal system. The weight of condensation on the outside wall peaked at 60 min after starting the AirSeal system and was maintained thereafter. However, the Heated group demonstrated a lower trend of the condensation volume than the Unheated group (p = 0.0676. Figure 2e). The accumulated condensation volume on the outside wall was significantly smaller in the Heated group than in the Unheated group (271.8 ± 26.4 vs. 353.6 ± 33.4 g, respectively; p = 0.0238. Figure 2f).

**Table 1:** Patient characteristics.

	Heated group	Unheated group	*p* value
	n = 19	n = 11	
Age (year)	74.0 (67, 80.2)*	77.0 (67.0, 80.2)*	n.s
Body weight (kg)	51.8 (48.3, 55.4)*	57.0 (50.0, 61.2)*	n.s
Location (C/A/T)	7/10/2	2/7/2	n.s
T (cT1–2/T3–4)	8/11	8/3	n.s
N (cN0/N+)	10/9	2/9	n.s
M (cM0/M+)	18/1	10/1	n.s
surgical procedures (ICR/RHC)	14/5	5/6	n.s
Lap-approach	19	11	n.s

*The data were described as interquartile range.

## Discussion

A dry surgical field is required to conduct precise and sophisticated surgery, which contributes to good outcomes especially in oncological surgery. The AirSeal system is a unique insufflation device that can flow carbon dioxide into the abdomen at a high flow rate without a mechanical valve on the access port. It creates and maintains stable pneumoperitoneum during surgery. However, condensation that forms on the access port sometimes drips onto the surgical field, making it wet. The wet condition of the surgical field impairs surgeons’ comfortable performance of precise surgery. Therefore, a dry surgical field must be maintained during laparoscopic and robotic surgery. The experimental results of this study revealed the mechanism of how condensation, which eventually drips onto the surgical field, initially develops on the access port. One of the unique features of the AirSeal system is its tri-lumen insufflation tube. This tube delivers gas to create pneumoperitoneum, evacuates smoke, and creates an air seal barrier on the top of the access port. The air seal barrier is established by the recirculated gas with the moisture from the abdominal cavity through the body of the AirSeal system, and this air seal barrier (a non-mechanical valve) is capable of maintaining stable pneumoperitoneum without excessive intra-abdominal pressure. However, the recirculated moist gas creates condensation inside the tri-lumen tube by cooling below body temperature. The accumulated condensation develops into water droplets in the tri-lumen tube, and these droplets flow into the inside of the access port. The condensation on the access port is created directly from the moist gas in the abdominal cavity because the carbon dioxide gas from the tanks decreases the objective temperature of the access port below the temperature of the abdominal cavity. The condensation then becomes water droplets on the outside wall of the access port, and these droplets sometimes drip onto the surgical field.

This study revealed that the condensation and water droplets attached to the access port can be reduced by heating the tri-lumen tube. Heating the tube can suppress the temperature difference between the influx and efflux of the recirculated moist gas. This reduced temperature difference suppresses the formation of condensation and droplets within the tube that might otherwise flow to the inside of the access port. As a result, the total volume of the condensation attached to the inside wall of the access port is reduced by heating the tri-lumen tube. Moreover, artificially heated gas can suppress the cooling of the access port. This might reduce the condensation attached to the outside wall of the access port.

The heating effect of carbon dioxide gas has been reported in many papers, as has the effect of humidification. Birch et al.^6^ reported the detailed effects of heating the insufflation tube in their systemic review. They reviewed 22 qualified randomized controlled trials in which the insufflation tube was heated with or without humidification during laparoscopic surgery. The main outcomes of those studies were the core temperature, pain score, morphine consumption, hospital stay, recovery time, lens fogging, and operative time. The authors concluded that there was no clear evidence for the usefulness of heated gas insufflation, with or without humidification. Most of those trials involved relatively minor surgery, such as laparoscopic cholecystectomy, laparoscopic gastric bypass, and hysterectomy^7–10^. Even in a double-blinded trial of colonic resection, a major surgery, no clinical benefits of warming and humidification of the insufflation gas were observed with respect to postoperative pain and the early inflammatory cytokine response^11^. In the present study, heating the tri-lumen tube did not affect the patients’ core temperature, which is consistent with the statement of the European consensus guideline that “the clinical benefits of warmed humidified insufflation lead to slightly higher core body temperature”^12^. However, our goal in the present study, which was totally different from the above-mentioned studies, was to improve the condition of the surgical field to facilitate comfortable performance of precise surgery by heating the insufflation tube as a solution for the wet condition associated with use of the AirSeal system. And also, in this study there were no adverse events during the observation.

None of the patients who underwent heating of the insufflation tube developed adverse events in this study. The limitations of this study include the small number of enrolled patients and the single-institutional design. However, we reduced the variable of surgical procedures as much as possible. The laparoscopic procedures in this study were determined to reduce the technical variables of surgery: it started from medial approach to perform lymph node dissection using electric cautery of monopolar and ultrasonic scalpel. Within the 2 h of the study, ileocolic artery was cut and medial approach was mostly completed. In this study, the heating silicon cover wrapped on the tube can warm only unsterile part of the tube because of the non-sterile silicon cover. If the heating silicon cover of the device can be sterilized, the insufflator gas would be warmed more effectively because of warming the full length of the tube. We are currently in the development stage of creating a heating device to warm the insufflation tube. Some insufflator devices have a heating system to warm the insufflation gas within the insufflation system. However, the AirSeal system and some other insufflator systems have no heating system to warm the insufflation gas. An insufflation gas heating system for universal use with all insufflators is needed. The heating device should be able to sterilize and resist water for clinical use. Therefore, we will continue to develop an improved universal heating device for insufflator tubes.

## Methods

In this study, the ethical principles of the declaration of Helsinki were followed. The study protocol was approved by the ethics committee of Fukuoka University Hospital (approval number 19-8-02). Informed consent for study enrollment was obtained from each patient.

### Measurement of condensation volume on access port using experimental simulator

The heating device was developed by Kawai COOPERATION in Nagoya. The parts of the device consist of the main body, the silicon cover with heating wire and the power cord. The device has the digital temperature control function with thermostat. The mechanism of heating was based on the electric resistance heating. The silicon cover can hold the tube which has the diameter of approximately 1.5–4 cm.

**Figure 3:**
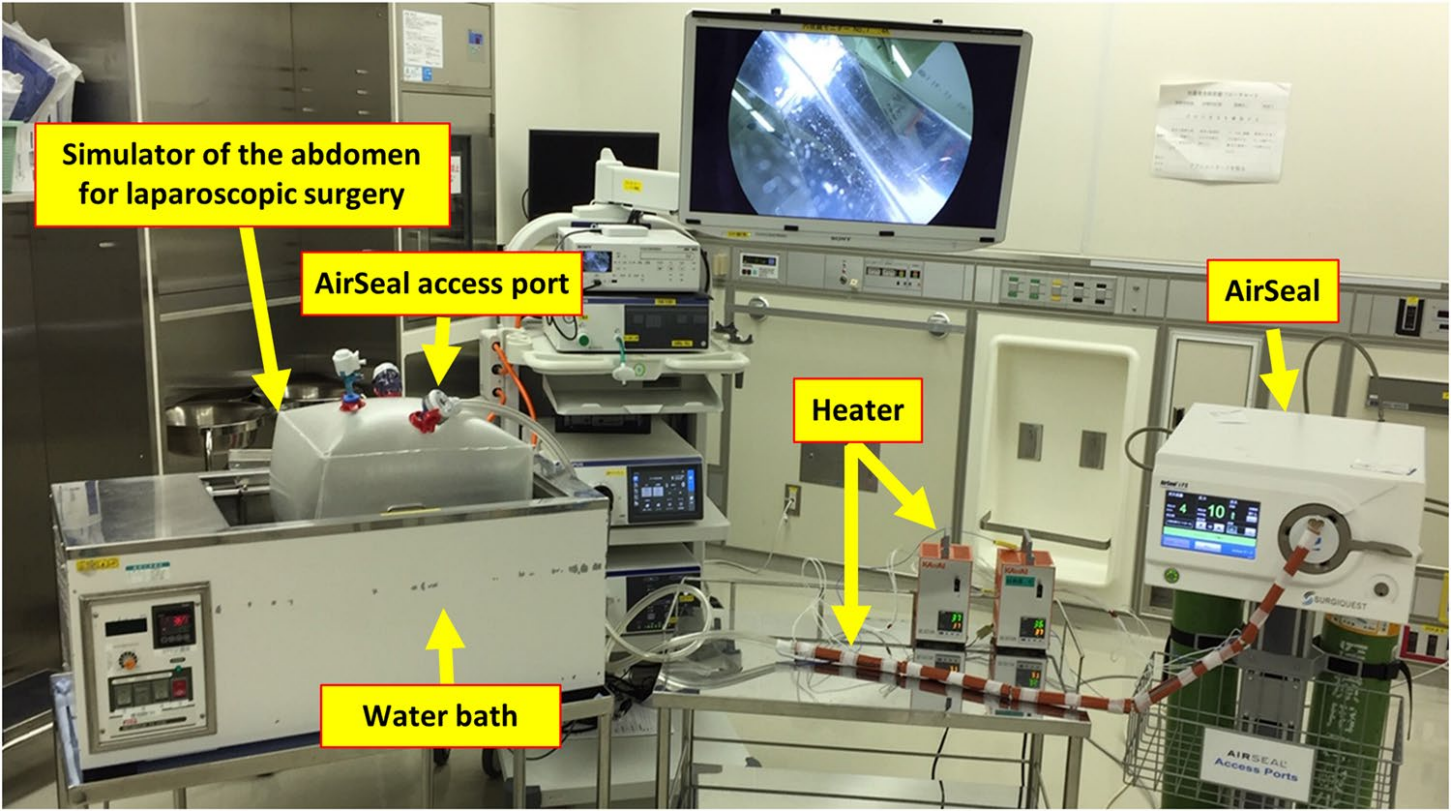
Experiment using a simulator to determine how condensation forms on the access port. The experimental simulator was floated on a water bath warmed to 37 °C, mirroring the abdominal cavity. The access port of the AirSeal was placed on the simulator, and the tri-lumen tube was connected from the body of the AirSeal system to the access port. The AirSeal system was switched on, and insufflation was started at a flow rate of 40 L/min and pressure of 10 mmHg. A silicone rubber heater was wrapped around the tri-lumen tube.

An experimental simulator was used to elucidate the relationship between the volume of water droplets attached to the access port and the temperature of the tri-lumen tube connected to the body of the insufflator (Fig. 3). The simulator had six holes for inserting the ports. The simulator was floated in a large thermostatic water bath warmed to 37 °C, and 100 mL of water warmed to 37 °C was poured into the simulator to create moisture for mirroring the intraperitoneal space. The access port of the AirSeal was placed on the simulator, and the tri-lumen tube was connected from the body of the AirSeal system to the access port. The AirSeal system was switched on, and insufflation was started at a flow rate of 40 L/min and pressure of 10 mmHg. From ten minutes after starting insufflation to maintain a steady state, the condensation and water droplets attached to the inside and outside of the wall of the access port were wiped with the small gauze every 10 min. The weight of the gauze was measured by the weight scale before and after wiping. The condensation and droplets volume were determined by the calculation as “the condensation and droplets volume = the weight of the gauze after wiping—the weight of the gauze before wiping”. The condensation volume was measured in three different temperature conditions. The tri-lumen tube was heated to 37 °C in the Heated group, cooled to 4 °C in the Cooled group, and maintained at room temperature (no heating or cooling) in the Control group. The condensation volume was measured under each condition.

### Intraoperative assessment of the effect of heating the insufflation tube

Patients undergoing laparoscopic colectomy were randomly divided into the Heated and Unheated groups. After starting insufflation with the AirSeal system, the weight of the condensation attached to the inside or outside of the access port was measured every 30 min for 2 h using a scale. The intra-abdominal humidity and the body temperature were also recorded at these same time points.

### Statistical analysis

Data are shown as mean ± standard error of the mean in line charts and as interquartile range on box plot graphs. The patients’ characteristics were statistically analyzed using the Wilcoxon test or the chi square test. Differences in the experimental and clinical studies of condensation were assessed by one-way analysis of variance (ANOVA) or repeated-measures ANOVA among the three groups. When one-way ANOVA was applied, the Tukey–Kramer honestly significant difference test was used to identify significant differences between the groups. Significant differences were determined when p < 0.05. The statistical analysis was conducted using JMP 14.0 software (SAS Institute, Cary, NC, USA).

### Supplementary Information

Supplementary Figure 1.

Supplementary Legends.

Supplementary Video 1.

Supplementary Video 2.

Supplementary Video 3.

## Data Availability

The datasets generated during and/or analyzed during the current study are available from the corresponding author on reasonable request.

## References

[1] Bucur P, Hofmann M, Menhadji A, Abedi G, Okhunov Z, Rinehart J (2016). Comparison of pneumoperitoneum stability between a valveless trocar system and conventional insufflation: A prospective randomized trial. Urology..

[2] Claroni C, Morettini L, Tola G, Covotta M, Forastiere E, Torregiani G (2022). Valveless trocar versus standard pneumoperitoneum insufflation system in minimally invasive surgery: Impact on postoperative pain. A systematic review and meta-analysis. J. Laparoendosc. Adv. Surg. Tech. A..

[3] Shahait, M. *et al*. Improved outcomes utilizing a valveless-trocar system during robot-assisted radical prostatectomy (RARP). *JSLS*. **23**(1), e2018.00085 (2019). 10.4293/JSLS.2018.00085 PMC6364705 30740014

[4] Sroussi J, Elies A, Rigouzzo A, Louvet N, Mezzadri M, Fazel A (2017). Low pressure gynecological laparoscopy (7mmHg) with AirSeal((R)) System versus a standard insufflation (15mmHg): A pilot study in 60 patients. J. Gynecol. Obstet. Hum. Reprod..

[5] Buda, A. *et al*. Low-pressure laparoscopy using the AirSeal system versus standard insufflation in early-stage endometrial cancer: A multicenter, retrospective study (ARIEL study). *Healthcare (Basel)*. **10**(3), 531 (2022). 10.3390/healthcare10030531 PMC8953067 35327010

[6] Birch DW, Dang JT, Switzer NJ, Manouchehri N, Shi X, Hadi G (2016). Heated insufflation with or without humidification for laparoscopic abdominal surgery. Cochrane Database Syst. Rev..

[7] Klugsberger B, Schreiner M, Rothe A, Haas D, Oppelt P, Shamiyeh A (2014). Warmed, humidified carbon dioxide insufflation versus standard carbon dioxide in laparoscopic cholecystectomy: A double-blinded randomized controlled trial. Surg. Endosc..

[8] Saad S, Minor I, Mohri T, Nagelschmidt M (2000). The clinical impact of warmed insufflation carbon dioxide gas for laparoscopic cholecystectomy. Surg. Endosc..

[9] Hamza MA, Schneider BE, White PF, Recart A, Villegas L, Ogunnaike B (2005). Heated and humidified insufflation during laparoscopic gastric bypass surgery: Effect on temperature, postoperative pain, and recovery outcomes. J. Laparoendosc. Adv. Surg. Tech. A..

[10] Nelskyla K, Yli-Hankala A, Sjoberg J, Korhonen I, Korttila K (1999). Warming of insufflation gas during laparoscopic hysterectomy: Effect on body temperature and the autonomic nervous system. Acta Anaesthesiol. Scand..

[11] Sammour T, Kahokehr A, Hayes J, Hulme-Moir M, Hill AG (2010). Warming and humidification of insufflation carbon dioxide in laparoscopic colonic surgery: A double-blinded randomized controlled trial. Ann. Surg..

[12] Neudecker J, Sauerland S, Neugebauer E, Bergamaschi R, Bonjer HJ, Cuschieri A (2002). The European Association for Endoscopic Surgery clinical practice guideline on the pneumoperitoneum for laparoscopic surgery. Surg. Endosc..

