# Supplementary figures and images for: Effect of heating insufflation tube of AirSeal system on laparoscopic surgery
Source: Sci Rep. 2024 Jan 5;14:646. doi: 10.1038/s41598-023-50321-y (PMC10770151; doi:10.1038/s41598-023-50321-y)

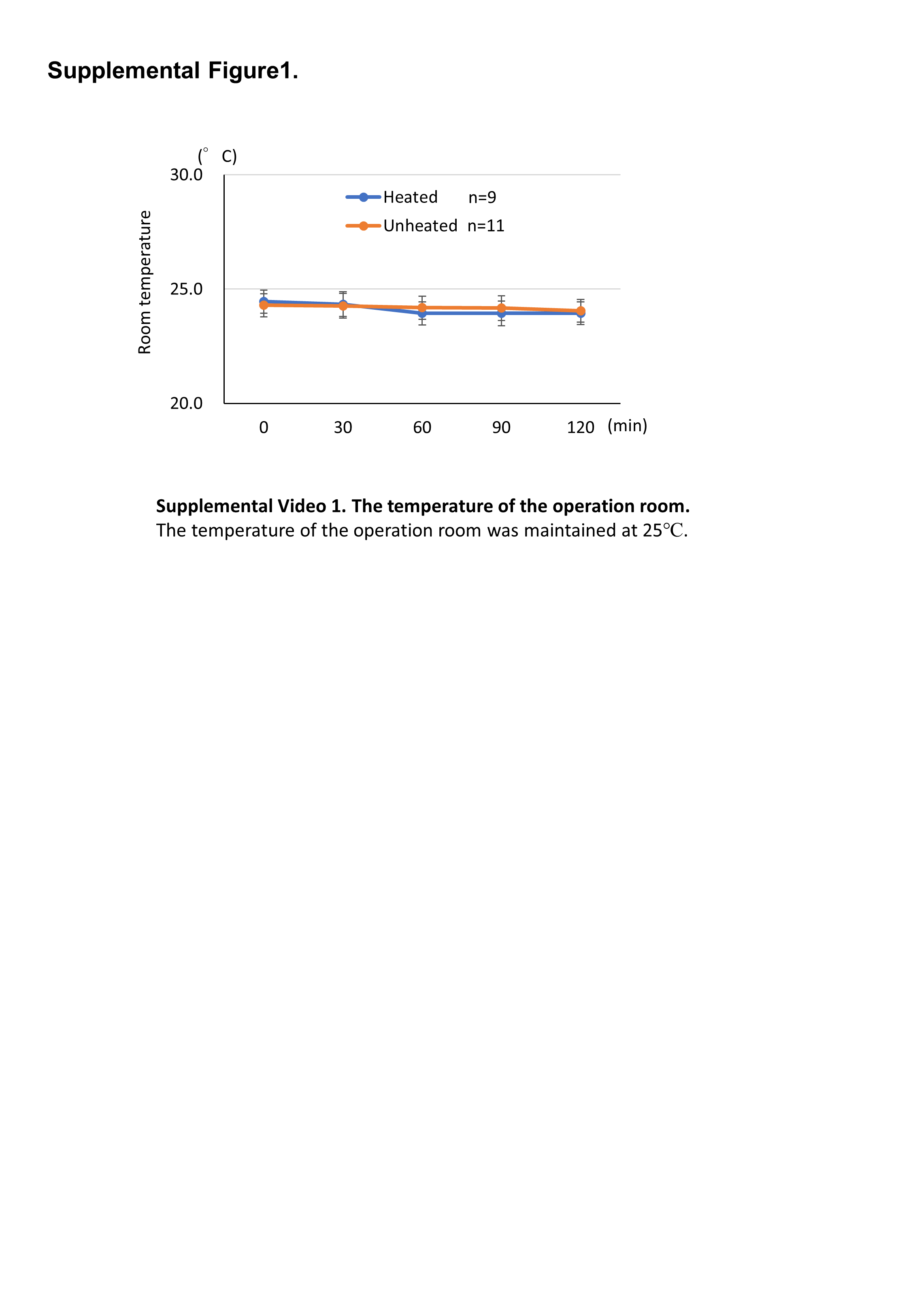

Supplement: Supplementary file 1 — Supplementary Figure 1. [file 41598_2023_50321_MOESM1_ESM.tif]
