# Supplementary material for: Effect of heating insufflation tube of AirSeal system on laparoscopic surgery
Source: Sci Rep. 2024 Jan 5;14:646. doi: 10.1038/s41598-023-50321-y (PMC10770151; doi:10.1038/s41598-023-50321-y)
Supplement: Supplementary file 2 — Supplementary Legends. [file 41598_2023_50321_MOESM2_ESM.docx]

Supplemental Video 1. Mechanism of condensation and droplet formation

Video 1a: Condensation was produced in the tri-lumen tube by cooling the moist gas recirculated to create the air seal cap.

Video 1b: The accumulated condensation changed to water droplets in the tri-lumen tube, and the droplets flowed into the inside of the access port.

Video 1c: The access port was cooled by the cooled insufflation gas, and the abdominal moist gas changed to condensation on the outside wall of the access port. The accumulated condensation changed to droplets that sometimes dripped onto the surgical field.
